# Recurrence rate with use of intraoperative Mitomycin C versus Conjunctival Autograft following pterygium excision

**DOI:** 10.12669/pjms.306.5191

**Published:** 2014

**Authors:** Quratulain Paracha, Mohammad Ayoob, Zafar Dawood, Sajid Ali Mirza

**Affiliations:** 1 Dr. Quratulain Paracha, FCPS, Assistant Professor, Ziauddin University Hospital, Keamari, Karachi, Pakistan.; 2 Dr. Mohammad Ayoob, FCPS, Assistant Professor, Ziauddin University Hospital, Keamari, Karachi, Pakistan.; 3 Dr. Zafar Dawood, FCPS, Associate Professor, Ziauddin University Hospital, Keamari, Karachi, Pakistan.; 4 Dr. Sajid Ali Mirza, FCPS, Professor. Ziauddin University Hospital, Keamari, Karachi, Pakistan.

**Keywords:** Conjunctival Autograft, Mitomycin C, Pterygium

## Abstract

***Objective*** ***s*** ***: ***To determine the recurrence rate following Conjunctival Autograft versus Mitomycin C for pterygium excision.

***Methods: ***Fifty Patients in this Randomized Clinical Trial who underwent pterygium excision from July 2013 to October 2013 at Department of Ophthalmology, Ziauddin University Hospital, Keamari, Karachi were included. All patients underwent detailed ophthalmic examination before surgery. Few drops of lidocaine were instilled, subconjunctival xylocaine 2% was injected. The pterygium was then excised from bulabar conjunctiva and peeled off from the corneal surface. Mitomycin C was applied to bare sclera in group A and Conjunctival autograft taken from superior bulbar conjunctiva of same eye was sutured to the bare sclera in group B. Data for pterygium recurrence was collected and analyzed using SPSS version 17.

***Results: ***Among the 50 patients operated 64% (n=32) were male and 36% (n=18) female. Their age ranged from 28 -58 years with mean age 44.8yrs. Right eye was affected in 54% (n= 27) patients and left in 46% (n= 23). In group “A” (intraoperative MMC) conjunctival granuloma was noted in 1(4%), pterygium recurrence 4(16%) and ocular irritation was experienced by 5 (20%) patients. In group “B” (CAG) graft retraction was seen in 2(8%), patients, 1(4%) patients experienced persistent redness over the grafted tissue and pterygium recurrence was seen in 2(8%) patient. All of them were followed at day 1, week1, week 4 and week 12.

***Conclusion: ***Both Conjunctival Autograft and Mitomycin C are effective in reducing the recurrence of pterygium but CAG gives better cosmetic results, the only drawback with it is the duration of the procedure.

## INTRODUCTION

Pterygium is a conjunctival disorder which usually occurs with increasing age. It arises in the interpalpaberal aperture with nasal aspect of palpaberal conjunctiva affected most frequently. Rarely it occurs both on the nasal and temporal aspect simultaneously. It is progressive condition, which often extends to involve peripheral cornea causing astigmatism and at times can block the visual axis. It has reported prevalence of 2% to 7% worldwide.^1^ Although its pathogenesis is not yet clear but ultraviolet radiation is supposed to be the strongest predisposing factor for its development besides hot warm climate, dry eye etc. Recently its been suggested that mutations in P53 gene on chromosome 17 may be responsible for it.^2^Excessive outdoor exposure put males more at risk and hence gender wise it occurs twice as frequently in males than female.^3^

The presentation could be either with watering, red eye, foreign body sensation, visual impairment or cosmetic disfigurement although asymptomatic cases are not uncommon. Anti-inflammatory agents and lubricants are given for symptomatic relief but they do not cause regression of the pterygium. 

The standard treatment for pterygium is surgical excision with bare sclera technique which is associated with recurrence as high as 24-89%.^4^ Therefore adjunct treatment options are in use to reduce this recurrence. Among these are use of beta radiation, conjunctival autograft, amniotic membrane grafting and mitomycin C. Recently 5 fluorouracil has also been used to reduce the recurrence.^5^

The recurrence of pterygium could occur due to fibroblast proliferation as part of healing process following conjunctival excision. Another hypothesis is that loss of limbal stem cell barrier function allows conjunctiva to grow over cornea.^2^ Therefore adjunct treatment aims at inhibiting the fibroblast proliferation or covering the bare sclera with a tissue of similar properties.

Mitomycin C is an antineoplastic antibiotic agent that selectively inhibits the synthesis of DNA, cellular RNA and protein, hence affects cellular proliferation for a long time. It can be used preopratively, intraoperatively or postoperatively. Single preoperative use of low dose MMC is safer than pre and postoperative application. The usual dose for intraoperative use varies from 0.01 to 0.04% for 3min to 5 minutes. Increasing the duration and concentration of MMC may be associated with complications like scleromalacia, corneal perforation, glaucoma, iritis, punctuate keratopathy.^6^ However there is 6.7% to 22.5% recurrence reported with use of intraoperative MMC in national studies*.* ^3^^,^^7^

Conjunctival autograft (CAG) as an adjunct to pterygium excision is a safe rocedure because limbal stem cells in donor tissue yields better healing of conjunctival and corneal tissue and also acts as a barrier to fibroblast proliferation. All this results in better cosmesis postoperatively. The only disadvantage with CAG is it is technically more demanding and time consuming. The reported recurrence with CAG varies from 5% to 9.09%.^3^^,^^8^^,^^9^

The aim of our study was to establish the procedure for pterygium with minimum recurrence and better cosmesis at our centre.

## METHODS

The study was conducted at department of Ophthalmology, Ziauddin University Hospital, Keamari, Karachi from July 2013 to October 2013. The patients with primary pterygium with recurrent inflammation, impaired vision or cosmetic disfigurement were selected randomly for pterygium excision with either intraoperative MMC or conjunctival autograft. While those with recurrent prerygium, glaucoma, conjunctival scarring or dry eyes were excluded. All patients were allocated to two groups “A & B” at random and were operated as day case procedure. All the patients were explained regarding expected outcome with the use of MMC or Conjunctival Autograft. The detailed examination of anterior and posterior segments were done by the surgeon and recorded on the proforma.

The Ethics Review Committee of the Ziauddin University has given the approval and informed written consent was taken from all participants.

**Fig 1:**
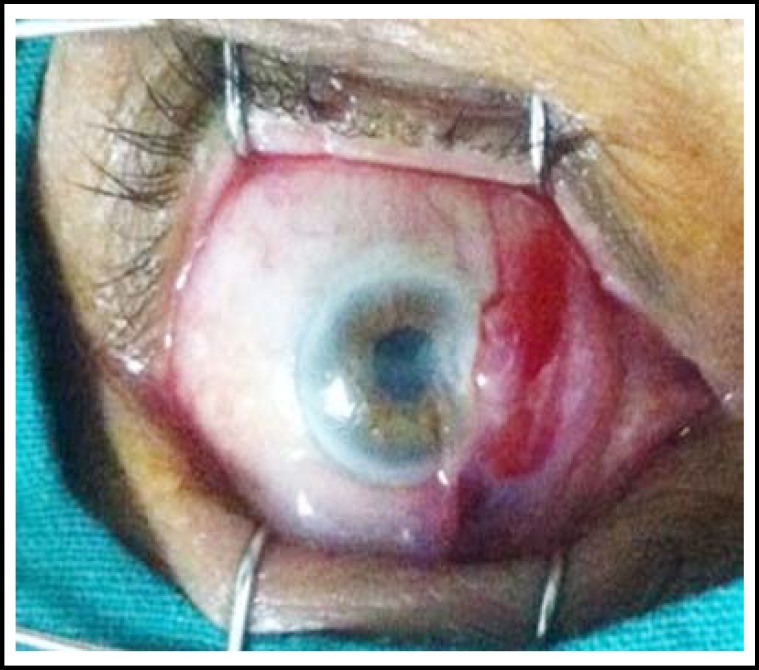
Peroperative: subconjunctival xylocaine 2% injected

**Fig 2:**
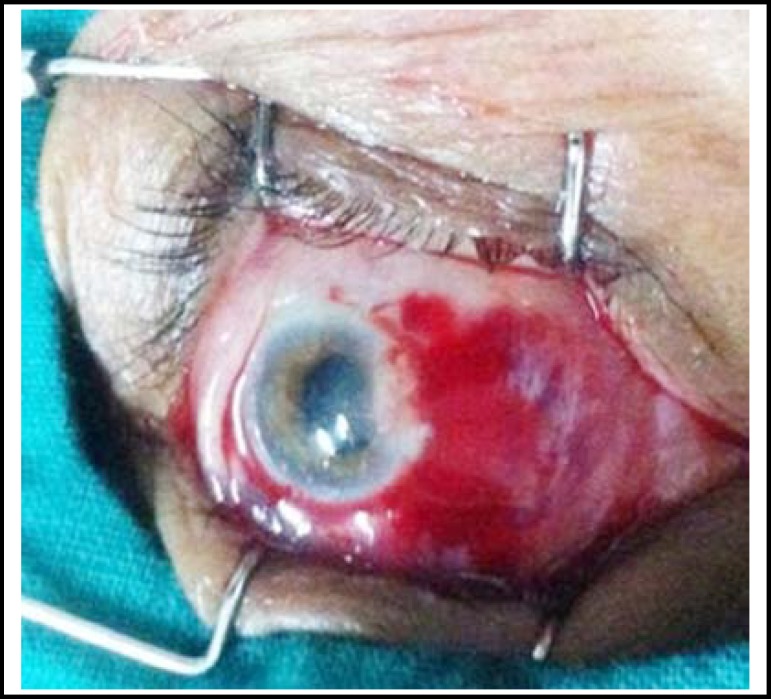
Peroperative: Bare Sclera to apply MMC

**Fig 3:**
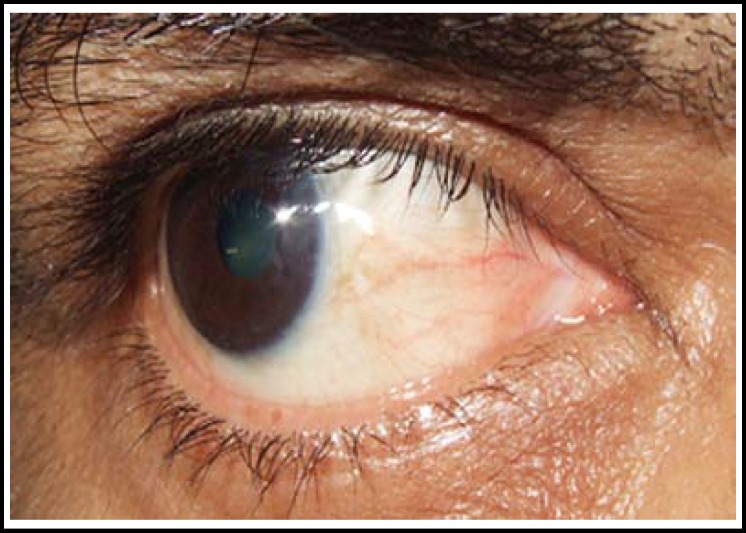
6 weeks postoperative CAG (nasal pterygium)

**Fig 4:**
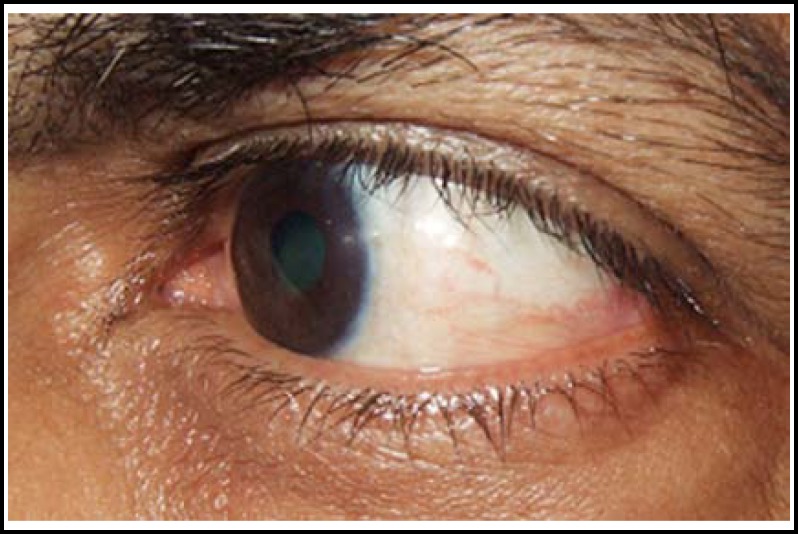
3 Months postoperative CAG (temporal pterygium)

All cases were done under local anaesthesia. A few drops of lidocaine were instilled and then 1 cc xylocaine 2% was injected subconjunctivaly in bed of pterygium.(Fig 1) The pterygium was then lifted from the body by giving stab incisions on its either side through the conjunctiva, excised from bulbar conjunctiva and peeled off the corneal surface, removing the tenon’s capsule and episcleral from underneath.(Fig 2) In group “A” cotton bud soaked in 0.02% MMC was applied to bare sclera for 2 minutes and then it was rinsed with ringer’s lactate solution. In group “B” The defect size was measured using the Castroviejo caliper and then Conjunctival Autograft was dissected from superior bulbar site after giving subconjunctival xylocaine. The graft was taken and implanted over bare sclera with limbal site facing the limbus and secured with 10/0 nylon sutures.(Fig 3&4) Subconjunctival antibiotic and steroid was given to all patients and eye pad was applied for 24 hours. Postoperatively topical antibiotic steroid combination was given for six weeks.

The follow up was done on day 1, week 1, week 4 and week 12. At each visit patient was examined for corneal and conjunctival wound healing. Any sign of recurrence was noted, which is defined as fibrovascular re-growth reaching or crossing the limbus. All surgeries were performed by the single surgeon.

## RESULTS

Fifty patients with primary pterygium were operated, among those 64% (n=32) were male and 36% (n=18) female. Their age ranged from 28 -58 yrs with mean age 44.8yrs. Right eye was affected in 54% (n= 27) patients and left in 46% (n= 23). Table 1 All the patients underwent thorough preoperative work up including visual assessment, refraction, anterior and posterior segment examination. There was no intraoperative complication noted in either group.

In group “A” patients where intraoperative MMC was used, conjunctival granuloma was noted in 1(4%) patient within 4 weeks, he was a poorly controlled diabetic patient and granuloma was excised later. While pterygium recurrence was observed in 4(16%) patients. Ocular irritation was experienced by 5 (20%) patients in first postoperative week till the wound was healed.

In group “B” postoperatively graft retraction was seen in 2(8%) patients probably due to its undersize but as no fibrovascular growth developed, therefore patients were simply observed. 1(4%) patient experienced persistent redness over the grafted tissue and pterygium recurrence was seen in 2(8%) patient. The donor site healed smoothly at its own. The sutures were removed at second postoperative follow up in all patients. Patients with intraoperative MMC showed greater recurrence than those who received conjunctival Autograft. (p=0.667) {Table 2}

**Table: 1:** Patient Data

	Laterality		Site of Pterygium		Gender	
	right	left	nasal	temporal	male	female
Group “**A**”	14	11	25	0	15	10
Group “**B**”	11	14	24	01	18	07

**Table: 2:** Pterygium Recurrence

	Group “**A**”	Group”**B**”
Recurrence	4(16%)	2(8%)

## DISCUSSION

This study was carried out to establish a procedure with minimum recurrence and better cosmesis following pterygium excision. In our study there was male preponderance over females as seen in a national study conducted by Kamil Z.^3^ Another study conducted by Khan N shows 63% male and 37% female patients of pterygium.^10^

The conjunctival auto grafting was first described by Kenyon et al. in 1985 and reported a recurrence rate of 5.3%^11^ The recurrence rate is reported as 4.6% in a study by Narsani AK et al. in 2013.^9^ In 2005 Fahmi et al. reported 13.3% recurrence with limbal CAG.^12^ Another study shows 5% recurrence with use of CAG.^3^ The recurrence rate of 7.69% is reported by Narsani AK in 2008.^9^ In our study we experienced a recurrence of 4% which is comparable. One patient in our series who had CAG for first eye, when presented with pterygium in second eye he opted for same surgical technique because of better cosmesis.

Mitomycin C has been used as subconjunctival injections preoperatively^13^, topical drops postoperatively for a week or two,^14^ but single intraoperative use is safer. In our study we applied low dose MMC for short duration i.e. 0.02% for only 2 minutes, this not only reduces the rate of recurrence but also the complication rate. According to Rubinfeld RS et al. complications are mainly related to the uncontrolled and prolonged use of MMC by the patients.^15^ In study by Kamil Z et al.^3^ 22.5% was the recurrence rate with adjunct use of intraoperative MMC while16.13% recurrence was noted By Narsani AK^9^. We found a recurrence rate of 16% in our series. Postoperative complications like scleral necrosis, corneal melting was not observed in our series of patients.

## CONCLUSION

Both Conjunctival Autograft and Mitomycin C are effective in reducing the recurrence of pterygium but CAG gives better cosmetic results. 

## Authors Contribution:

***Quratulain Paracha:*** Patient selection, conceived, designed, performed surgeries, manuscript writing. ***Mohammad Ayoob:*** Data analysis and manuscript review. ***Zafar Dawood:*** Patient selection, data collection. ***Sajid Ali Mirza: ***Manuscript review.

## Notes

***Funding:*** None.
